# Protection conferred by a recombinant Marek’s disease virus that expresses the spike protein from infectious bronchitis virus in specific pathogen-free chicken

**DOI:** 10.1186/1743-422X-9-85

**Published:** 2012-05-04

**Authors:** Xiaorong Zhang, Yantao Wu, Yezhen Huang, Xiufan Liu

**Affiliations:** 1 Laboratory of Animal Infectious Diseases, School of Veterinary Medicine, Yangzhou University, Yangzhou, 225009, PR China

**Keywords:** Infectious bronchitis virus, Spike protein, Marek’s disease virus

## Abstract

**Background:**

In many countries, the predominant field isolates of infectious bronchitis virus (IBV) have been classified as QX-like strains since 1996. However, no commercial vaccines that are specific for this type of IBV are currently available. Therefore, there is an urgent need to develop novel vaccines that prevent QX-like IBV infection.

**Results:**

A recombinant Marek’s disease virus (MDV), rMDV-S1, that expresses the S1 subunit of the spike (S) protein from the QX-like infectious bronchitis virus (IBV) was constructed by inserting the IBV S1 gene into the genome of the CVI988/Rispens strain of MDV. Specific pathogen-free (SPF) chickens that were vaccinated with rMDV-S1 were protected when challenged with the QX-like IBV. They were observed to have mild clinical signs of disease, a short virus-shedding period and low mortality. Additionally, the rMDV-S1 conferred full protection to chickens against virulent MDV, as did the CVI988/Rispens strain.

**Conclusions:**

Our results demonstrate that rMDV-S1 is an effective and promising recombinant vaccine for the prevention of QX-like IBV infection.

## Background

Infectious bronchitis (IB) is an acute, contagious disease of both layer and broiler chickens. This disease is caused by infectious bronchitis virus (IBV) and has respiratory signs. In hens infected with IBV, decreased egg production and quality are often observed. Some IBV strains are nephropathogenic, and infection with these strains can result in interstitial nephritis and mortality [1]. IBV encodes four major structural proteins: the spike (S) protein, the nucleocapsid (N) protein, the membrane (M) protein, and the envelope (E) protein. The S protein of IBV is initially translated into a precursor glycoprotein that is cleaved post-translationally to form two subunits, S1 and S2 [2]. Most of the virus-neutralizing antibodies target the S1 protein [3]. Currently, both modified live and inactivated vaccines are commercially available [1]. Despite the intensive vaccination efforts, outbreaks of IBV frequently occur due to the persistence of different IBV serotypes. These serotypes arise from point mutations, insertions, deletions, and RNA recombination [3]. Multiple distinct genetic groups of IBV have been reported, but since 1996, the predominant field isolates in China have been classified as QX-like (LX4-type) [4-8]. QX-like IBVs are circulating in almost all of the countries in Europe, and these variants are frequently associated with cases of nephritis and false layer syndrome in chickens [9-14]. Clearly, QX-like IBVs are a serious threat to the poultry industry. However, no vaccines against QX-like IBVs are commercially available. Therefore, there is an urgent need to develop vaccines that can prevent QX-like IBV infection.

Marek’s disease (MD) is a transmissible, malignant T-cell lymphoma in chickens caused by Marek’s disease virus (MDV) [15]. MDV strains are classified into the following three serotypes: Gallid herpesvirus 2 (MDV serotype 1), Gallid herpesvirus 3 (MDV serotype 2), and Meleagrid herpesvirus 1 (MDV serotype 3/herpesvirus of turkeys) [16]. All of the pathogenic strains of the virus are included in serotype 1, and they range from highly virulent to weakly virulent strains. These strains, such as CVI988/Rispens, may be attenuated by extensive serial passage in tissue culture, with loss of pathogenic properties but retention of immunogenicity. The CVI988/Rispens strain provides good protection against challenge even with the newly discovered highly virulent pathotype of MDV [1].

MDV has several ideal characteristics that make it useful as a vector for recombinant vaccine. MDV infection results in a persistent infection, and because it is transmitted cell to cell, the virus is not susceptible to maternal antibodies. Furthermore, the virus has a large genome with several regions that are not essential for viral replication, which are suitable for the insertion of foreign genes [17]. In the present study, a recombinant MDV (rMDV) expressing the S1 protein from QX-like IBV (rMDV-S1) was constructed, and the protective efficacy of the virus as a vaccine against both IBV and MDV in SPF chickens was investigated.

## Results

### Construction of the transfer vector

**Figure 1:**
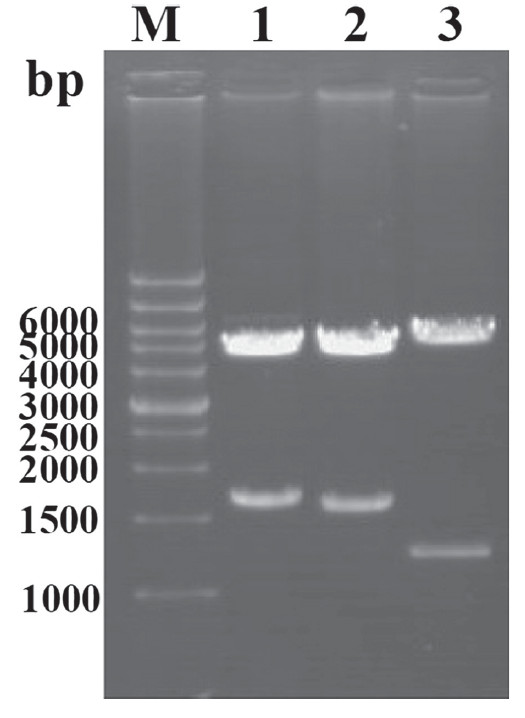
** Identification of the transfer vector by restriction enzyme digestion analysis.** M: DNA Marker; Lane 1: pUP-S1-DOWN digested with * Avr * II and * Not * I; Lane 2: pUP-LTR-EGFP-S1-DOWN digested with * Not * I; Lane 3: pUP-LTR-EGFP-S1-DOWN digested with *Hin*d III.

To construct the recombinant MDV, a transfer vector called pUP-LTR-EGFP-S1-DOWN was constructed by inserting the IBV S1 gene, the LTR promoter from reticuloendotheliosis virus (REV) and an EGFP expression cassette into the pUP/DOWN plasmid. The construction of the transfer vector was verified by restriction enzyme digestion analysis (Figure 1) and DNA sequencing.

### Construction and characterization of the recombinant viruses

**Figure 2:**
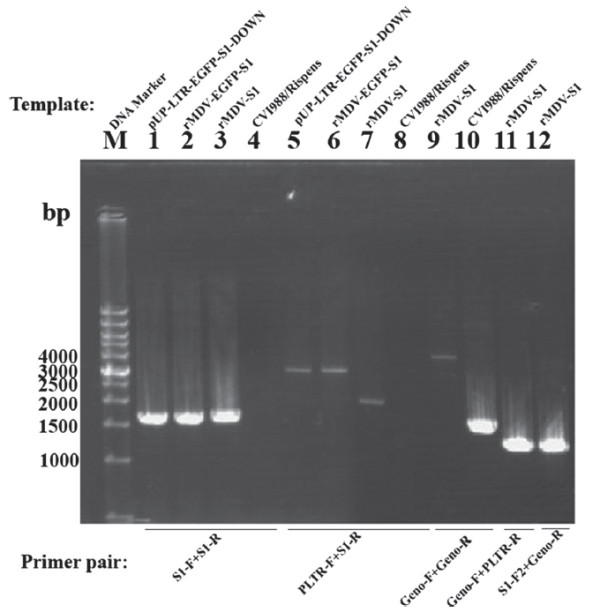
** Verification of the structure of the rMDVs by a series of PCR reactions.** The S1 gene could be detected from both rMDV-EGFP-S1 (lane 2) and rMDV-S1 (lane 3) with primers S1-F and S1-R. The transfer vector pUP-LTR-EGFP-S1-DOWN was used as positive control (lane 1), and CVI988/Rispens DNA was used as a negative control (lane 4). The correct orientation of the inserted genes was further confirmed with the primers PLTR-F and S1-R (lanes 6 and 7). The transfer vector pUP-LTR-EGFP-S1-DOWN was used as a positive control (lane 5), and CVI988/Rispens DNA was used as a negative control (lane 8). The incorporation of the foreign gene in the correct site of rMDV-S1 genome was also verified with the following three primer pairs: Geno-F and Geno-R (lane 9), Geno-F and PLTR-R (lane 11), and S1-F2 and Geno-R (lane 12). CVI988/Rispens DNA was used as a negative control (lane 10).

First, we analyzed the recombinant rMDV-EGFP-S1 for the expression of the EGFP protein by fluorescence microscopy. When all the plaques were visibly fluorescent, the EGFP gene between the two loxP sites was removed by Cre-mediated recombination. The recombinant viruses, rMDV-EGFP-S1 and rMDV-S1, were verified by a series of PCR reactions. The results indicated that recombination occurred at the expected locus in the CVI988/Rispens genome, and all inserted elements were arranged as intended (Figure 2).

**Figure 3:**
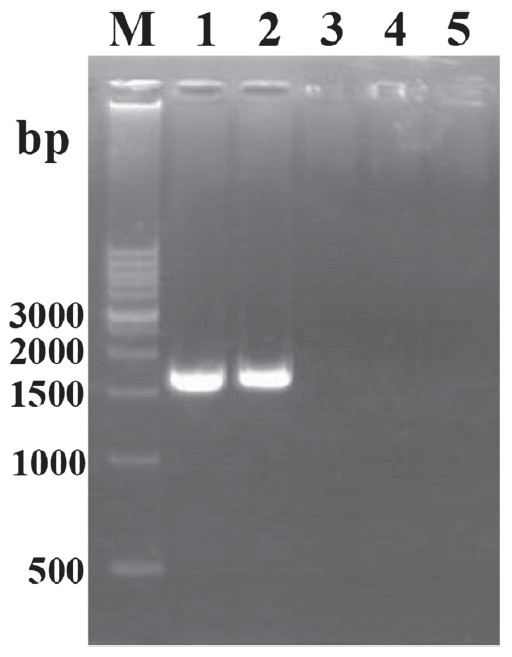
** RT-PCR verification of S1 gene expression in rMDV S1-infected CEFs.** M: DNA Marker. PCR template used independently in reactions 1 ~ 5: pUP-LTR-EGFP-S1-DOWN (positive control); cDNA reverse-transcribed with RNA isolated from rMDV-S1-infected CEFs; RNA isolated from rMDV-S1-infected CEFs; cDNA reverse-transcribed from RNA isolated from CVI988/Rispens-infected CEFs; RNA isolated from CVI988/Rispens-infected CEFs.

**Figure 4:**
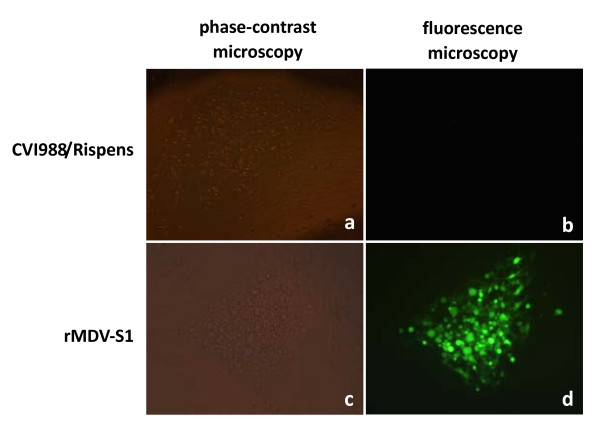
** Detection of S1 protein expression by IFA.** Plaques of rMDV-S1 were immunologically stained with an IBV-specific antibody, whereas plaques of CVI988/Rispens were not.

To confirm the expression of the S1 protein of rMDV, an RT-PCR assay was used to detect S1 gene mRNA transcription in rMDV-S1-infected chicken embryo fibroblasts (CEFs) using the S1-F and S1-R primers. An expected 1.7-kb PCR product could be amplified from mRNA isolated from the rMDV-S1-infected CEFs but not from CVI988/Rispens-infected CEFs (Figure 3). CEFs infected with the rMDV-S1 were stained with chicken anti-IBV serum. Plaques from the rMDV-S1 were stained by the chicken anti-IBV serum, whereas plaques from the CVI988/Rispens virus were not (Figure 4).

The rMDV-S1 virus was genetically stable after 30 sequential passages in CEFs.

### Induction of the IBV antibody response

**Figure 5:**
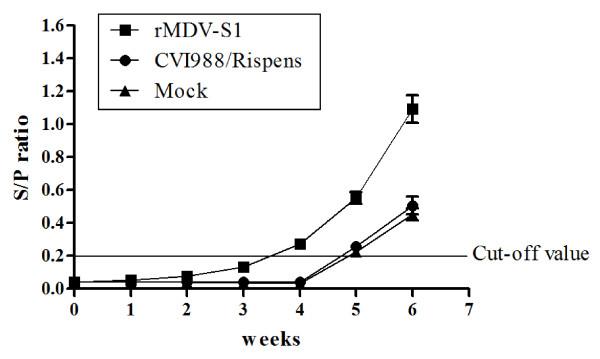
** Antibody responses to IBV after vaccination and challenge.** The vaccination time-point (1-day-old) was recorded as 0 w. Chickens were challenged at 4 w post-vaccination.

Figure 5 indicates the change in the levels of IBV-specific antibodies following the inoculation of SPF chickens with rMDV-S1, CVI988/Rispens, or phosphate buffered saline (PBS) that were subsequently challenged with IBV. No specific, anti-IBV antibodies were detected in chickens that were inoculated with CVI988/Rispens or PBS. The rMDV-S1 vaccinated chickens were observed to have a low-level antibody response until 4 weeks post-vaccination, but after IBV challenge, the antibody level increased more rapidly compared with the control group. These results indicated that the rMDV-S1 virus could induce an IBV-specific immune response in vaccinated chickens.

### Protection against IBV challenge

Following challenge with the IBV, CK/CH/JS/06II strain, 70% (14/20) of the chickens in the rMDV-S1-vaccinated group had no signs of IBV, and 95% (19/20) of the chickens survived. In contrast, all of the chickens in the control group and in the CVI988/Rispens-vaccinated group developed serious clinical symptoms and were observed to have a higher mortality after challenge with IBV, CK/CH/JS/06II strain (Table 1). All deaths occurred within 3 to 5 days after challenge. A chi-squared (*χ*^*2*^) test to compare the morbidity and mortality between each treatment group indicated that there was a significant difference (*p <* 0.05) between the rMDV-S1-vaccinated group and the PBS mock-vaccinated group.

**Table 1:** Morbidity and mortality of vaccinated chickens after challenge with the IBV CK/CH/JS/06II strain

**Groups**	**Morbidity**	**Mortality**
**rMDV-S1**	6/20 ^a^	1/20 ^a^
**CVI988/Rispens**	20/20 ^b^	6/20 ^b^
**PBS**	20/20 ^b^	6/20 ^b^

***Note:*** The criteria for morbidity were as follows: (1) wheezing or dyspnea, (2) swinging of the head, and (3) feathers erected and dullness. The presence of any of these criteria can be used to evaluate morbidity. The morbidity and mortality among the four experimental groups are indicated with different lowercase letters and differ significantly based on *χ*^2^ analysis (*p* < 0.05).

From days 2 to 12 after challenge, tracheal swabs were collected for virus isolation from 10 randomly selected chickens from each group. The results indicated that the virus could be recovered from the rMDV-S1-vaccinated group from days 4 to 8 and from the two control groups from days 4 to 10. The *χ*^*2*^ test indicated that at specific time points, there was a significant difference between the rMDV-S1-vaccinated group and the PBS mock-vaccinated group (*p <* 0.05) in the number of tracheal swabs that tested positive for the virus (Table 2). The virus detection rate from the kidneys of the chickens in the rMDV-S1-vaccinated group was lower compared with the control groups. There was a highly significant difference (*p <* 0.01) in the number of kidneys that tested positive for the virus between the rMDV-S1-vaccinated and the PBS mock-vaccinated groups (Table 2). Together, these data indicate that in the rMDV-S1-immunized group, the virus-shedding period was shorter than that of the control groups.

**Table 2:** Virus isolation from tracheal swabs or from the kidneys of vaccinated chickens after challenge with the IBV CK/CH/JS/06II strain

**Groups**	**Isolation of virus**^**★**^						
	**Days post-virus challenge**						
	2*	4*	6*	8*	10*	12*	14**
**rMDV-S1**	0/10 ^a^	10/10 ^a^	4/10 ^a^	1/10 ^a^	0/10 ^a^	0/10 ^a^	2/19 ^a^
**CVI988/Rispens**	0/10 ^a^	10/10 ^a^	10/10 ^b^	6/10 ^b^	3/10 ^b^	0/10 ^a^	14/14 ^b^
**PBS**	0/10 ^a^	10/10 ^a^	10/10 ^b^	6/10 ^b^	4/10 ^b^	0/10 ^a^	14/14 ^b^

* Virus isolation from trachea swabs.

** Virus isolation from kidney.

^★^ Virus isolation from the trachea or the kidney from the four experimental groups, as indicated with different lowercase letters, differed significantly based on *χ*^2^ analysis (*p* < 0.05).

Table 3 indicates that after challenge, the rate of increase in the bodyweight of chickens in the CVI988/Rispens-vaccinated and PBS mock-vaccinated groups was significantly lower than in the rMDV-S1-vaccinated (*p <* 0.01) group.

**Table 3:** Bodyweight changes of vaccinated chickens after challenge with the IBV CK/CH/JS/06II strain

**Groups**	Bodyweight (g)*		
	**1-day-old**	**Pre-challenge**	**Post-challenge**
**rMDV-S1**	49.5 ± 0.8 ^a^ (n = 20)	259.6 ± 10.9 ^a^ (n = 20)	433.0 ± 29.8 ^a^ (n = 19)
**CVI988/Rispens**	49.4 ± 0.7 ^a^ (n = 20)	256.6 ± 11.4 ^a^ (n = 20)	395.7 ± 48.7 ^b^ (n = 14)
**PBS**	49.5 ± 1.1 ^a^ (n = 20)	258.8 ± 10.9 ^a^ (n = 20)	391.6 ± 50.9 ^b^ (n = 14)

* Mean ± SE. Bodyweight among the four experimental groups, as indicated by different lowercase letters, differed significantly based on *χ*^2^ analysis (*p* < 0.05).

**Figure 6:**
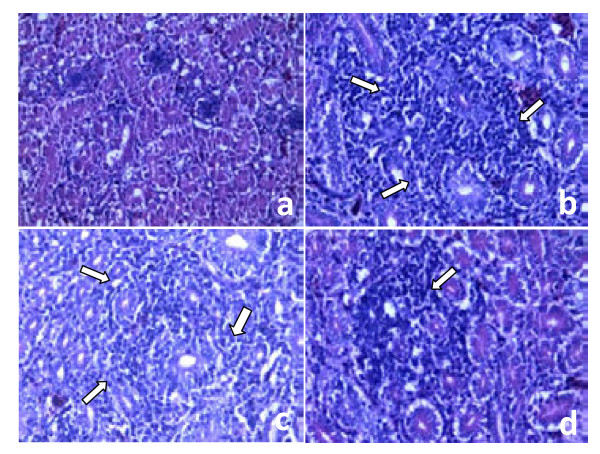
**Examination of histological lesions in the kidneys of vaccinated chickens at 14 d post-challenge (H&E staining, Magnification 400×).** (a) Healthy control, (b) PBS control, (c) CVI988/Rispens and (d) rMDV-S1. (b) and (c) were observed to have more severe nephritis compared with (d). The arrows indicate the regions with infiltration of round cells.

Necropsy of the deceased chickens revealed the typical pathology of IB. The disease was restricted to the upper respiratory tract and the kidneys of infected chickens and characterized by the presence of mucus in the upper respiratory tract as well as gray and mottled kidneys. On day 14 post-challenge, microscopic kidney lesions were visible in all chickens. In the chickens from the control group and the CVI988/Rispens-immunized group, the lesions were characterized by massive infiltration of round cells. In the rMDV-S1-immunized group, more moderate lesions were observed compared with the control groups (Figure 6).

### Protection against MDV challenge

To determine the protective efficacy of rMDV-S1 vaccination against the virulent RB1B strain of MDV, chickens were vaccinated and then challenged with RB1B MDV 7 days after vaccination and examined for signs of MD and tumor development for 6 weeks. The chickens vaccinated with rMDV-S1 and those vaccinated with CVI988/Rispens did not show any clinical signs of MD and had no gross or histopathological tumors. These data indicate that rMDV-S1 vaccination could confer protection against MDV. 90% (18/20) of the chickens in the unvaccinated control group died of MD, and the remaining 2 chickens had histopathological MD lesions present at the time of necropsy (Table 4).

**Table 4:** Protective efficacy of the rMDV-S1 virus against MDV RB1B challenge

**Groups**	**Clinical signs**	**Mortality**	**Gross lesions**	**Histopathological lesions**	**PI (%)**
**rMDV-S1**	-	0% (0/20)	0% (0/20)	0% (0/20)	100
**CVI988/Rispens**	-	0% (0/20)	0% (0/20)	0% (0/20)	100
**control**	+	90% (18/20)	95% (19/20)	100% (20/20)	

***Note:*** One-day-old SPF chickens were vaccinated with either the rMDV-S1 or CVI988/Rispens virus and challenged 7 days later with the MDV RB1B strain. Clinical signs and mortality were observed for 6 weeks, and the chickens that died during challenge and those that were euthanized at the end of the observation period were necropsied and subjected to gross and histopathological examination. Chickens with gross tumor lesions were considered positive for histopathological lesions.

## Discussion

Antigenic variation among IBV strains is common [3,18-28]. Due to the regular emergence of antigenic variants, the occurrence of disease and the use of vaccines will vary by geographical region. According to phylogenetic analysis by Han *et al.*[5], most of the recently described IBV isolates were from 9 distinct genetic groups, and the predominant group was the QX-like strain [6,29]. In China, the commonly used IBV vaccine strains, such as H120, H52 and M41, are all Mass-type vaccines, and the S1 gene sequence similarity is low compared with the currently circulating IBV strains [19]. This result could explain why the increasing number of vaccination failures. Thus, developing novel vaccines against QX-like IBVs is necessary [5,19,23].

The construction of a recombinant virus vector-based vaccine is a good option to develop IBV vaccines that are specific to new IBV serotypes. Fowlpox viruses that express the IBV S1 gene have been reported previously [30-32], and all of these vaccines have been described to confer protection against viral challenge with homotypic IBV strains and some heterotypic IBV strains in SPF chickens. However, the protective efficacy that is conferred by recombinant fowlpox viruses could be diminished in commercial chickens due to the presence of maternal antibodies against fowlpox virus [33,34]. In fact, fowlpox vaccines are widely used in commercial chickens, which hinder the application of recombinant vaccines that are based on fowlpox virus vectors. Compared with other viral vectors, the CVI988/Rispens has several unique advantages. The CVI988/Rispens is the most effective MD vaccine obtained thus far [35]. Additionally, the CVI988/Rispens can persistently infect chickens, which results in the continuous expression of inserted, heterologous antigens [17]. Finally, the CVI988/Rispens can be used to inoculate one-day-old chickens to establish an early immunity.

Although the rMDV-S1 vaccinated group induced a low level antibody response until 4 weeks post-vaccination, but the level of antibody increased more rapidly compared to the control groups following IBV challenge. Additionally, chickens vaccinated with rMDV-S1 were observed to have a shorter virus-shedding period and a greater increase in bodyweight. These results suggested that rMDV-S1 immunization could induce an efficient primary immune response that was specific to the expressed S1 protein. When the chickens were challenged with IBV, the primed B cells could rapidly differentiate into plasma cells, thus permitting the production of higher titers of IBV-specific circulating antibody within a shorter period of time. This finding is important for the establishment of an early-stage active immune response against IBV infection.

## Conclusions

We constructed a recombinant MDV, called rMDV-S1, that expressed the S1 protein of a QX-like IBV. Vaccination with rMDV-S1 conferred good protection in SPF chickens against both IBV and MDV challenge. Taken together, rMDV-S1 is a promising vaccine candidate for the control of infection by QX-like IBVs: the major IBV genotype currently circulating in many countries.

## Materials and Methods

### Viruses, SPF chicken embryos and chickens

The CVI988/Rispens strain of MDV was used as the vector for the construction of the recombinant virus, and the virulent RB1B strain of MDV was used as the challenge virus. MDVs were propagated in secondary CEFs in Medium 199 supplemented with 3% newborn calf serum (Thermo Fisher Scientific).

The QX-like IBV strain CK/CH/JS/06II was used for S1 gene [GenBank: EU031526] amplification and as the challenge virus. The challenge dose was determined in a previous experiment in SPF chickens. This dose (2 × 10^3.5^ EID50 per chicken) could induce clinical signs, including death, beginning on day 2 post-challenge.

SPF chicken embryos were purchased from Merial-Vital Laboratory Animal Technology Co., Ltd. (Beijing, China). SPF chickens were hatched from SPF embryos and reared in negative-pressure isolators. Feed and water were available *ad libitum*. This study was conducted in strict accordance with the recommendations given in the Guide for the Care and Use of Laboratory Animals of the National Research Council. The animal care and use committee of Yangzhou University approved all experiments and procedures conducted on the animals (approval ID: SYXK (Su) 2007-0005).

### Construction of the transfer vector

**Figure 7:**
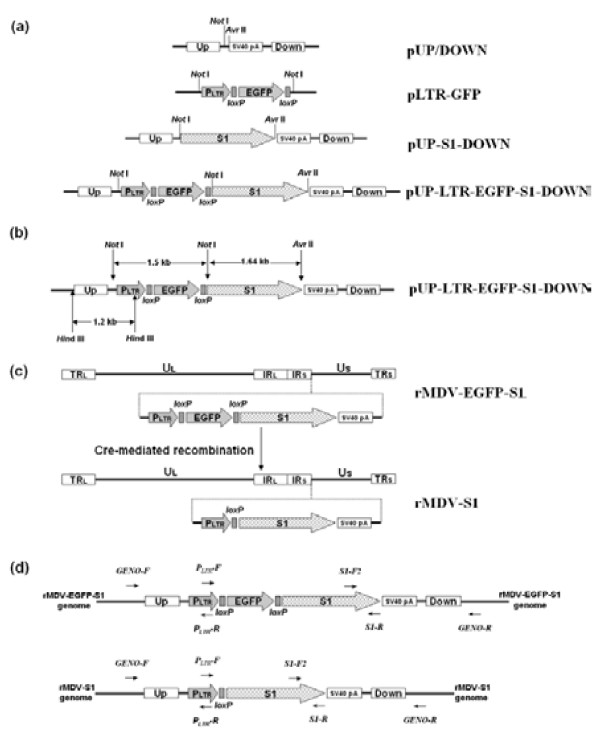
**Illustration of the construction of rMDV-S1.** (a) Construction of the transfer vector. (b) Identification pattern of the transfer vector by restriction enzyme digestion. (c) Steps for generating rMDV-S1. (d) Primer binding sites mapped to the genome of the rMDVs.

The plasmid pUP/DOWN that contained the homologous sequence to the IRS-US junction region of the CVI988/Rispens genome [GenBank: DQ530348], the plasmid pLTR-GFP that contained a loxP-flanked EGFP expression cassette and a LTR promoter from REV were constructed previously by Li *et al.*[36]. The S1 gene from the IBV strain CK/CH/JS/06II was amplified from genomic RNA with M-MLV reverse transcriptase and Phusion® High-Fidelity DNA Polymerase (New England BioLabs) using primers S1-F (5′-AAAGCGGCCGCGAGATGTTGGGGAAGTCACTG-3′) and S1-R (5′-ATTCCTAGGTTAGCCATAACTAACATAAGGGCAACT-3′). These primers were designed based on the S1 gene sequence of CK/CH/JS/06II. Artificial *Avr* II and *Not* I restriction sites (underlined) permitted the direct cloning of a 1641-bp fragment of the resulting amplicon into the pUP/DOWN vector. This resulted in the plasmid pUP-S1-DOWN. To produce the transfer vector pUP-LTR-EGFP-S1-DOWN containing an EGFP reporter gene, the plasmid pLTR-GFP was digested with *Not* I (New England BioLabs), and the LTR-EGFP expression cassette was cloned into the *Not* I-digested, pUP-S1-DOWN plasmid in the same orientation as the S1 gene (Figure 7a). The insertion of both the S1 gene and LTR-EGFP was verified by *Avr* II and *Not* I (New England BioLabs) digestion. The correct orientation of the insertion was confirmed by *Hin*d III (New England BioLabs) digestion, as illustrated in Figure 7b. The transfer vector was further confirmed by gene sequencing (GenScript, Nanjing, China).

### Construction of rMDV

The recombinant virus was produced by calcium phosphate-mediated transfection of secondary CEFs that were cultured in a 60-mm dish with 100 ng of the pUP-LTR-EGFP-S1-DOWN transfer vector and 10 μg of CVI988/Rispens genomic DNA [37]. The transfected CEFs were cultured at 37°C in 5% CO_2_ for 5–7 days until viral plaques were visible. Viral plaques that expressed EGFP were identified by fluorescence microscopy (Leica DM RIB). These plaques were subsequently scraped from the monolayer using a micropipette containing a small amount of 0.25% trypsin. The cells were then aspirated, trypsinized into a single cell suspension, and passaged repeatedly. This process was repeated until all of the visible plaques were fluorescent. The resulting intermediate recombinant virus rMDV-EGFP-S1 was then amplified in CEFs.

Genomic DNA from the rMDV-EGFP-S1 virus was prepared as described above, and the EGFP gene between the two loxP sites was excised with Cre recombinase (New England BioLabs), which ensured that the S1 gene was placed immediately downstream of the LTR promoter. The genomic DNA from rMDV-EGFP-S1, in which the EGFP gene had been excised, was used for subsequent transfection experiments to generate new recombinant viruses that contained no reporter gene. The resulting non-fluorescent rMDV, called rMDV-S1, was screened and purified as described above. The complete process is illustrated in Figure 7c.

### PCR Identification of rMDV-S1

Primers S1-F and S1-R were used to amplify the inserted S1 gene from rMDV-S1. To further verify the correct orientation of the S1 gene, the primer PLTR-F (5′-ATCGGCATCAAGAGCAGG-3′) was designed according the sequence of the LTR promoter in combination with the primer S1-R that would amplify a 2.0-kb DNA fragment that comprised part of the LTR promoter sequence and the entire S1 gene sequence if the recombinant virus was engineered correctly. To verify whether the recombination event occurred at the expected location in the genome of the CVI988/Rispens vector, four other primers, designated Geno-F (5′-GCGTAGCCAGACCGCACCA-3′), Geno-R (5′-GCCTGAATAAAGACCGACA-3′), PLTR-R (5′-ATTGGCTCAGTATGATAGTTC-3′) and S1-F2 (5′-TTGCCACGTCAAGGAAGC-3′), were designed. The binding site for Geno-F was located upstream of the left homologous arm (153275–153293 nt in the CVI988/Rispens genome), and Geno-R was located downstream of the right homologous arm (154958–154976 nt in the CVI988/Rispens genome). PLTR-R and S1-F2 were located in the LTR promoter and the S1 gene, respectively. A detailed PCR scheme is illustrated in Figure 7d.

CEFs were harvested, and the total cellular RNA was prepared using TRIzol reagent (Invitrogen) after 48 hours of infection with either the rMDV-S1 or CVI988/Rispens. Total RNA was treated with DNase I (New England BioLabs) to eliminate any genomic DNA contamination and was then reverse-transcribed using oligo (dT)_15_ primers (Takara, Dalian, China). Subsequently, the S1 gene was amplified with primers S1-F and S1-R as described above. A positive control reaction using the transfer vector pUP-LTR-EGFP-S1-DOWN as the amplification template was performed. Additionally, a reaction with DNase I-treated total RNA as the amplification template was included in the RT-PCR analysis to check for DNA contamination.

### Indirect immunofluorescence assay (IFA)

CEFs were grown in a 24-well tissue culture plate and infected with rMDV-S1 or the CVI988/Rispens (100 PFU/well) vector. After incubation for 48 h at 37°C, these cells were fixed for 15 min with methanol-acetone (1:1) and incubated for 30 min in the presence of a 5 μg/ml dilution of a chicken anti-IBV polyclonal antibody that was prepared via multiple immunizations of SPF chickens with the Mass-type IBV antigen (China Institute of Veterinary Drug Control, Beijing, China) and a 7.5 μg/ml dilution of fluorescein-conjugated rabbit anti-chicken IgY (H+L) (Thermo Fisher Scientific) at 37°C. After each step, the cells were washed three times for 5 min with PBS. The cells were overlaid with 90% glycerol containing 25 mg/ml 1,4 diazabicyclo[2.2.2]octane (Sigma-Aldrich). The binding of the fluorescein-conjugated, secondary antibodies was analyzed by fluorescent microscopy (Leica DM RIB).

### Genetic stability of rMDV

The rMDV-S1 was sequentially passaged in CEFs to determine the genetic stability of the virus. As described above, the genomic DNA from passages 5, 10, 15, 20, 25 and 30 were extracted and used as a template for amplification of the S1 gene. IFAs were also applied to confirm the expression of the S1 gene in each passage.

### Protective efficacy of rMDV vaccination against IBV

Sixty, one-day-old SPF white Leghorn chickens were divided into 3 groups with 20 chickens in each group. The chickens were raised in individual isolators under negative pressure. Two groups were inoculated subcutaneously on the back of the neck with 5 × 10^3^ PFU of rMDV-S1 or CVI988/Rispens. The third group was mock-injected with PBS. An additional 10 chickens were raised separately to serve as the healthy controls.

Serum was collected weekly after vaccination until the chickens were challenged. Pre-vaccination serum was also collected. Serum samples were assayed in single dilutions (1:500) using an Infectious Bronchitis Virus Antibody Test Kit (IDEXX, ME, USA) according to the manufacturer’s instructions. S/P ratios that were greater than 0.2 were considered positive.

Four weeks after immunization, chickens from each group were challenged by the ocular-nasal administration of 2 × 10^3.5^ EID50 of the IBV CK/CH/JS/06II strain in 200 μl PBS per chicken. Any clinical disease symptoms, including death, beginning on day 2 post-challenge were recorded. On days 2, 4, 6, 8, 10, and 12 post-challenge, tracheal swabs were collected and used for virus isolation from 10 randomly selected chickens from each group. Dead chickens were necropsied immediately, and the live chickens from each group were euthanized 14 days post-challenge. The kidney tissues from all chickens were collected for virus isolation and pathological examination. Each chicken was weighed at 1-day-old, pre-challenge and at two weeks post-challenge.

The tracheal swabs and 20% tissue suspensions were freeze-thawed three times and centrifuged at 1000 × g. After centrifugation, the resulting supernatant was used to inoculate the allantoic cavity of 10-day-old SPF chicken embryos. After 96 hours, the allantoic fluid was collected, and any pathological changes in the embryos were recorded. The samples were considered positive for IBV isolation if the embryos appeared to be congestive, dropsical or hemorrhagic or if they developed urate deposition [32]. Otherwise, the embryos were inoculated for three blind passages. Allantoic fluid from the chicken embryos was collected and analyzed using RT-PCR as described above.

The collected kidney tissues were fixed in 10% neutral buffered formalin. The tissues were processed by standard histological procedures, embedded in paraffin, and cut into 5-mm sections. All of the sections were stained with hematoxylin and eosin (H&E).

### Protective efficacy of rMDV vaccination against MDV

A total of 60 SPF chickens were used in this experiment. Twenty, 1-day-old SPF chickens were vaccinated with the rMDV-S1 or the CVI988/Rispens as described above and challenged intraperitoneally after 7 days with MDV, RB1B strain (500 PFU/chick). Twenty unvaccinated SPF chickens were used as the challenge controls. The chickens were examined for clinical signs of disease and mortality for 6 weeks after challenge. The chickens that died after challenge and those that were sacrificed at the end of the challenge period were subjected to gross and histopathological observations for MD lesions in the liver, kidneys, spleen, nerves, and skin [17]. The protection index (PI) was calculated by the following formula [38]:

(1) $$PI(\%)=\frac{\%\;MD\;positive\;in\;controls\;-\;\%\;MD\;in\;vaccine\;group}{\%\;MD\;positives\;in\;controls}\times 100$$

### Statistical analyses

The differences in morbidity, mortality and virus-shedding period from the tracheal swaps and kidneys between each group were compared using the *χ*^*2*^ test. The difference in the increase in body weight (means ± SE) between each group was analyzed by Student’s *t* tests. The increase in bodyweight was analyzed by ANOVA. All statistical analyses were performed using the SPSS software (version 17.0. SPSS Inc., IL, USA).

## Abbreviations

MD, Marek’s disease; MDV, Marek’s disease virus; IB, Infectious bronchitis; IBV, Infectious bronchitis virus; S, Spike protein; PI, Protection index; CEF, Chick embryo fibroblasts; IFA, Indirect immunofluorescence assay; PBS, Phosphate buffered saline..

## Competing interests

The authors declare that they have no competing interests.

## Authors’ contributions

XZ performed the laboratory experiments and drafted the manuscript. YW conceived the study, participated in the research design and coordination and helped to draft the manuscript. YH carried out the animal experiments. XL participated in the design of the study. All authors read and approved the final manuscript.
